# Fatal Toxoplasmosis in Red Kangaroos (*Macropus rufus*) in East China

**DOI:** 10.3390/pathogens14020202

**Published:** 2025-02-19

**Authors:** Haiyan Gong, Quan Wang, Yinghong Jin, Suoping Qiu, Zhaoguo Chen, Xiangan Han, Zongyan Chen, Wei Jiang

**Affiliations:** 1 Shanghai Veterinary Research Institute, Chinese Academy of Agricultural Sciences, Shanghai 200241, China; 2 Institute of Veterinary Medicine, Xinjiang Academy of Animal Sciences, Urumqi 830013, China; 3 Conghua Customs Comprehensive Technical Service Center, Guangzhou 510900, China

**Keywords:** red kangaroo, *Toxoplasma gondii*, toxoplasmosis, stray cats, east China

## Abstract

Background: As a ubiquitous apicomplexan parasite, *Toxoplasma gondii* causes huge economic losses and poses a great threat to the health of animals, including humans, worldwide. In some kangaroo species, *T. gondii* can be fatal. To date, little information is available on *T. gondii* infection in the red kangaroos in east China. At a zoo in east China, thirteen red kangaroos consecutively developed clinical signs from July to November 2016, resulting in the deaths of seven, three of which were analyzed in this study. Methods: In the present study, ascitic fluid, blood and samples from the brain, heart, liver, spleen, lung, kidney, and mesenteric lymph nodes of three dead red kangaroos were collected. The pathogen was explored through microscopic observation, nested PCR, immunofluorescence antibody test (IFAT), hematoxylin–eosin (HE) staining, and immunohistochemistry (IHC) staining, respectively. Meanwhile, the potential source of the infection was also investigated by testing the blood of stray cats in the zoo for *T. gondii* using nested PCR. Results: Three dead red kangaroos were subjected to a necropsy, and organisms resembling *T. gondii* were detected in their ascitic fluids under microscope. This infection was further confirmed by a nested PCR assay, which resulted in a successful amplification and sequencing of the 433 bp fragment of the *T. gondii* 5.8S rRNA gene in all the dissected tissues, including heart, liver, spleen, lung, kidney, lymph nodes, cecum, and brain, as well as in body fluids (blood and ascitic fluid). Furthermore, the tachyzoites were observed in the heart, liver, spleen, lymph nodes, cecum, and brain through IFAT and HE staining. Administration of classic drugs (sulfadiazine and pyrimethamine) against *T. gondii* significantly alleviated the clinical signs of the sick kangaroos. The possible source of this infection was traced to a native stray cat, as *T. gondii* DNA was detected in its blood. Conclusions: In the present study, lethal *T. gondii* infection in red kangaroos has been described for the first time in east China, highlighting the necessity and urgency for close and long-term surveillance of this parasite infection in captive animals. The same strain of *T. gondii* detected in kangaroos as that found in stray cats wandering in the same area emphasizes the importance of controlling stray cat populations to mitigate the risk of *Toxoplasma* transmission to other animals.

## 1. Introduction

*Toxoplasma gondii* is a worldwide zoonotic Apicomplexa parasite that infects warm-blooded animals, including birds, mammals, and even humans [1]. It causes huge economic losses to animal husbandry [2] and poses great threats to people, especially pregnant women [3]. Among the hosts, lemurs [4], Pallas’s cats [5], meerkats [6], and kangaroos [7] are very sensitive to this parasite infection. Interestingly, the risk of infection in kangaroos is associated with species, gender, and living environment. Higher infection rates were reported in small kangaroo species, in females, and in captive kangaroos (32–100%) [7] compared to those observed in big species, males, and wild ones (3.3–20%) [8,9]. After infection, some macropods display pathological changes such as pulmonary congestion, adrenal enlargement and reddening, pericardial and peritoneal effusion, multi-focal necrosis, etc. [10], whereas several dead kangaroos exhibit no apparent clinical symptoms or lesions attributable to toxoplasmosis [11], which might shelter the parasite from detection.

Cats, especially feral ones, that roam in the environment and mainly feed on prayed small mammals are more apt to be infected with *T. gondii*. As the definitive host of the parasite, cats facilitate the completion of the life cycle of *T. gondii* and its maintenance in nature, excreting oocysts in their feces that can survive for several months and up to one year [12]. Moreover, oocysts contained in water or in soil are considered as the main source for the transmission of the parasite [13]. With the boundless movement of cats, the oocysts are spread widely, which, in turn, enhances the likelihood of infecting intermediate hosts [14].

Kangaroos in China are all captive in zoos, which hints at their high susceptibility to *T. gondii* infection. Recently, several articles have reported a high infection rate and mortality of macropods in China, including albino red-necked wallabies, red-necked wallabies, red kangaroos [15], and eastern gray kangaroos in Henan province [16]. The genotypes isolated in these cases were TgRooCHn1 (atypical type III) [16], TgRooCHn2 (type II), TgRooCHn3 (type III) [11], and TgRooCHn4 (type II variant) [15]. However, little information is available regarding kangaroos in east China that are associated with *T. gondii* infection.

At a zoo in east China, two species of kangaroos were housed, 18 red kangaroos (*Macropus rufus*) and 24 gray kangaroos (*Macropus fuliginosus*). Both species were kept in the same enclosure and were fed with the same feed and water. All the kangaroos were dewormed annually, and no new kangaroos were introduced to the enclosure. However, stray cats were always observed roaming within the enclosure. From July to November in 2016, 13 red kangaroos consecutively developed clinical signs of illness, with a peak in cases occurring the beginning of August. The sick red kangaroos were treated with antibiotics (ceftriaxone or azithromycin), acetylspiramycin, or sulfadiazine (SDZ), respectively. However, these treatments failed to alleviate the clinical signs, and instead, the death of seven red kangaroos was registered. To identify the pathogen responsible for this infection, the carcasses of three female adult red kangaroos were immediately sent to our lab on the day they died for post-mortem examination and further analysis through nested polymerase chain reaction (PCR), immunofluorescent antibody test (IFAT), hematoxylin–eosin (HE), and immunohistochemistry (IHC) staining analysis. Simultaneously, an investigation was conducted on blood samples collected from native stray cats to identify the potential source of this infection. This study aims to provide evidence for the urgency for close, long-term monitoring of *T. gondii* infections in captive animals and effective control of stray cats.

## 2. Materials and Methods

### 2.1. Clinical Signs and Sample Collection from the Dead Kangaroos

After necropsy, ascitic fluids and blood samples as well as tissues, including the heart, liver, spleen, lung, kidney, mesenteric lymph nodes, cecum, and brain, were collected from three dead red kangaroos for further study. Simultaneously, the water and feed provided to the kangaroos were collected for further analysis.

### 2.2. Identification of T. gondii in the Tissues and Body Fluids Using Nested PCR

The tissues and liquids collected above were extracted for DNA using a DNA rapid extraction kit (UltraPure, Shanghai, China). Then, primers targeting the 5.8S rRNA gene of *T. gondii* were employed for amplifying the parasite DNA in the samples according to a protocol described elsewhere [17]. The resulting fragments were purified by an AxyPerpTM DNA Gel Extraction kit (Axygen, Union City, CA, USA) and subjected to sequencing at the Shanghai Invitrogen Biotechnology Company (Shanghai, China). The water and feed for the kangaroos were subjected to the same nested PCR analysis as described above.

### 2.3. IFAT Analysis of T. gondii in the Tissues

After paraffin embedding and slicing of the dissected tissues (performed by Wuhan Goodbio Technology Co., Ltd., Wuhan, China), the slides were dewaxed in xylene and anhydrous ethanol, followed by antigen repairing in EDTA antigen repair buffer (pH 8.0, Catalog No. G1206). Then, 3% BSA was added to the slides for 30 min at room temperature before the first antibody (anti-SAG1 antibody in mice, diluted 1:100 in PBS) incubation at 4 °C overnight. Alexa Fluor 488-labeled goat anti-mouse second antibody (diluted 1:300 in PBS) was employed for the reaction at room temperature for 50 min, followed by staining of the nucleus with DAPI. Finally, the slides were observed under a Nikon inverted fluorescent microscope (Nikon Corporation, Tokyo, Japan). All the solutions and antibodies were provided by Wuhan Goodbio Technology Co., Ltd., China.

### 2.4. HE and IHC Staining of the Tissues

As for the HE staining, the slides from different dissected tissues were, respectively, treated with Hematoxylin Staining Solution (Wuhan Guge Biological Technology Co., Ltd., Wuhan, China) according to the manual. In the IHC staining process, the samples on the slides were subjected to a similar process as described in the IFAT analysis with a few modifications. Before the blocking in BSA, the slides were incubated with a 3% H_2_O_2_ solution (H_2_O_2_:H_2_O = 1:9) in a dark environment for 25 min to deactivate the endogenous peroxidase. HRP-labeled goat anti-mouse second antibody (1:1, DAKO, Kyoto, Japan) was applied to the slides for 50 min, followed by color development using DAB agent (DAKO, Kyoto, Japan). After the staining of the nucleus with hematoxylin–Harris for 3 min, the slides were dehydrated in xylene and sealed for further observation.

### 2.5. Detection of T. gondii in Stray Cats by Nested PCR

A total of eight stray cats, roaming in the kangaroo enclosure and lacking detailed information, were captured by specialists from a stray cat shelter. One milliliter of blood with anticoagulant was collected from the brachiocephalic vein of each stray cat, preserved at 4 °C and then subjected to DNA extraction, followed by nested PCR amplification for the *T. gondii* 5.8S rRNA gene using the same method as described above. The stray cats were released after blood sampling.

### 2.6. Treatment of the Infected Kangaroos

Following the identification of *T. gondii* in the red kangaroos, each kangaroo (with or without clinical signs) was orally administered with a combination of SDZ at a dose of 50 mg/kg and pyrimethamine (PYR) at 1 mg/kg continuously for 5 days [18]. The treatment’s effectiveness was then evaluated in the sick kangaroos.

## 3. Results

### 3.1. Clinical Signs and Necropsy Findings

**Figure 1:**
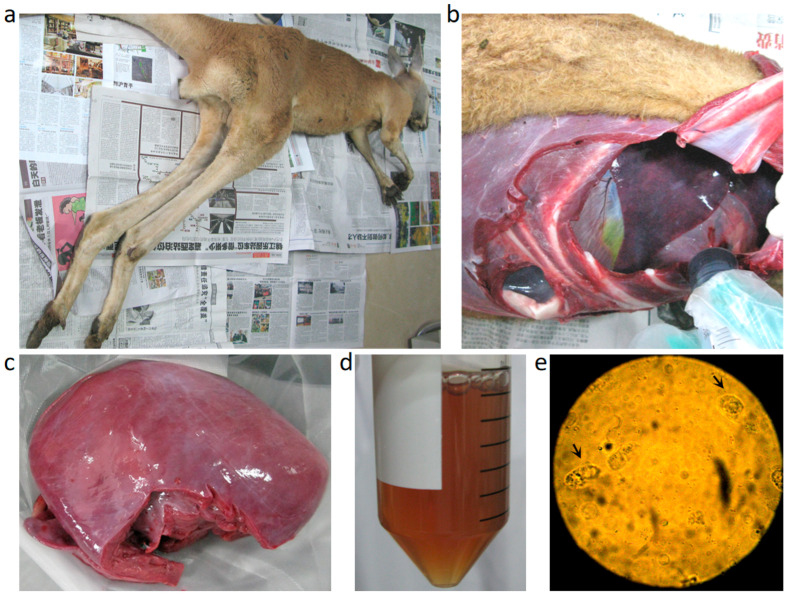
Necropsy findings. (**a**) General body appearance. (**b**) Abdominal cavity with ascitis. (**c**) Myocardial ischemia. (**d**) Ascitic fluid. (**e**) Cysts (arrows) presented in the ascitic fluid.

Thirteen red kangaroos exhibited clinical signs, representing approximately 31% (13/42) of the entire group. The mortality among the sick kangaroos was about 54% (7/13), including one juvenile and six adults (five females and one male). The sick red kangaroos exhibited anorexia, loss of body weight, and weakness in jumping. As shown in Figure 1a, the carcasses of the dead animals were clearly emaciated. Peritoneal effusion (Figure 1b) as well as ischemia in the heart (Figure 1c) were observed in the dead animals, and approximately 30 mL of ascitic fluids was collected (Figure 1d), which contained detectable *T. gondii* (Figure 1e).

### 3.2. Identification of T. gondii in the Different Tissues from the Dead Kangaroos Using Nested PCR

**Figure 2:**
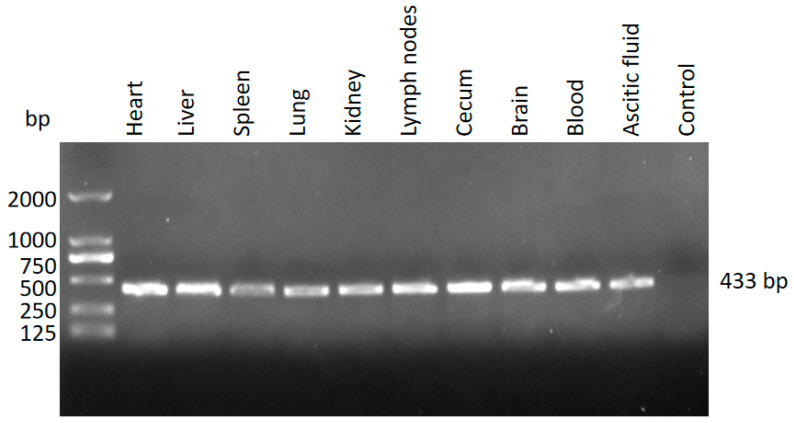
Detection of *T. gondii* in the different tissues using nested PCR.

Nested PCR amplification targeting the 5.8S rRNA gene of *T. gondii* was performed in various tissues (heart, liver, spleen, lung, kidney, mesenteric lymph nodes, cecum, and brain) and body fluids (blood and ascitic fluid). Positive signals were detected in all tested samples (Figure 2). The sequence of the amplicons matched 100% with that of the *T. gondii* clone CBa (accession number: JX456457.1), which had been previously identified in stray cats in Shanghai [17]. Simultaneously, the water and feed provided to the kangaroos were tested for contamination with *T. gondii* oocysts using nested PCR. The results were negative, ruling out these sources as potential contributors to the infection.

### 3.3. Identification of T.gondii in the Different Tissues from the Dead Kangaroos by IFAT

**Figure 3:**
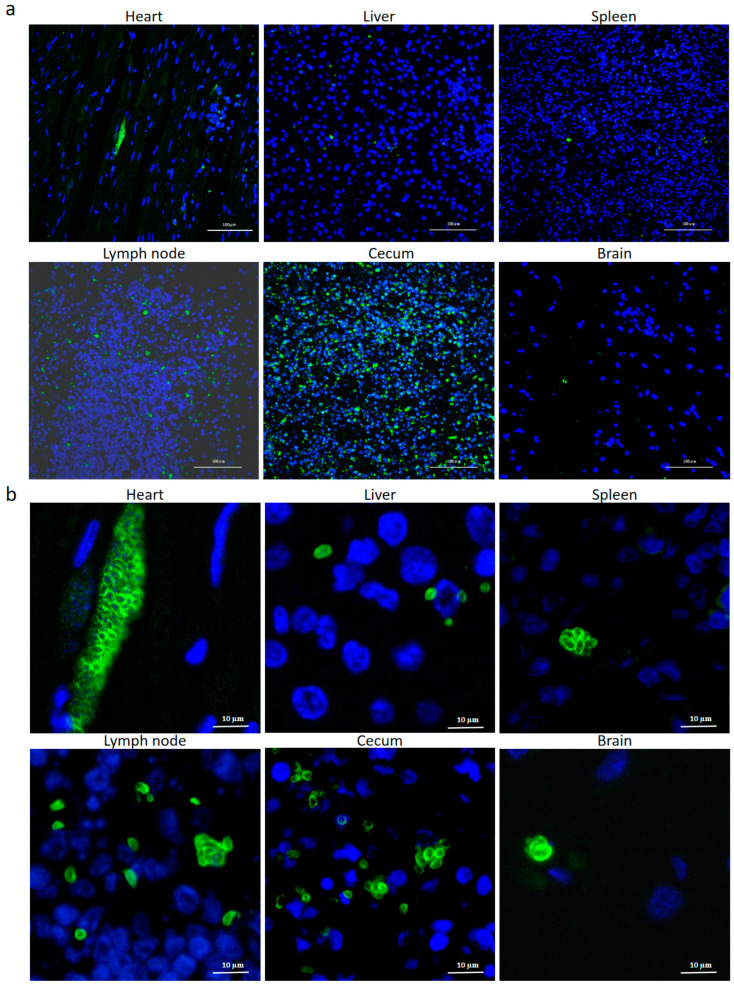
Detection of *T. gondii* in different tissues from the dead kangaroos by IFAT. The cysts of *T. gondii* in different tissues were observed under the microscope at 200× (**a**) and 400× (**b**) magnification. The nucleus and SAG1 protein were labeled with blue and green colors, respectively.

After labeling *T. gondii* with anti-SAG1 antibody, the tachyzoites were detected in the heart, liver, spleen, lymph nodes, cecum, and brain, with a widespread distribution in the cecum and lymph nodes (Figure 3a), while the fewest parasites were detected in the brain (Figure 3a,b).

### 3.4. Identification of T. gondii in Different Tissues from the Dead Kangaroos by HE and IHC Staining

**Figure 4:**
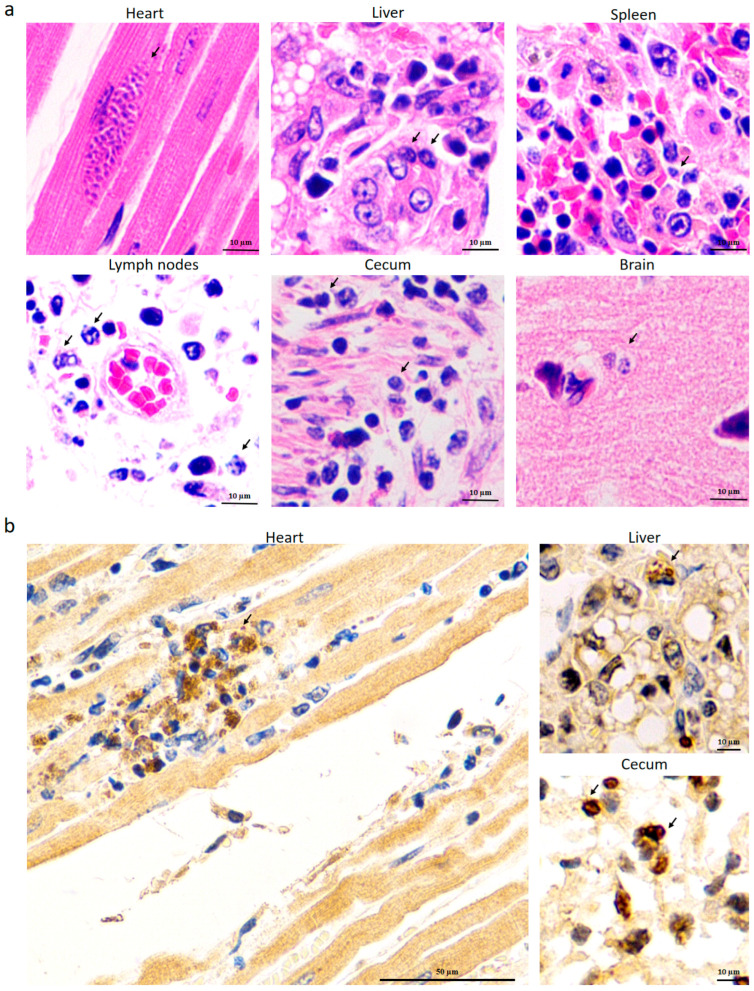
Detection of *T. gondii* in different tissues of dead kangaroos by HE staining (**a**) and IHC staining (**b**). The cysts are shown with arrows.

Consistent with the result of IFAT analysis, *T. gondii* was detected in the heart, liver, spleen, lymph nodes, cecum, and brain using HE staining (Figure 4a). IHC staining further distinguished the parasites from muscle nuclei by their characteristic brown color, as observed in the heart (left panel of Figure 4b). The tachyzoites were also detected in the liver and cecum (right panels of Figure 4b). In addition, the three dead kangaroos demonstrated similar clinical signs and pathological features.

### 3.5. Detection of T. gondii in the Blood of a Native Stray Cat

**Figure 5:**
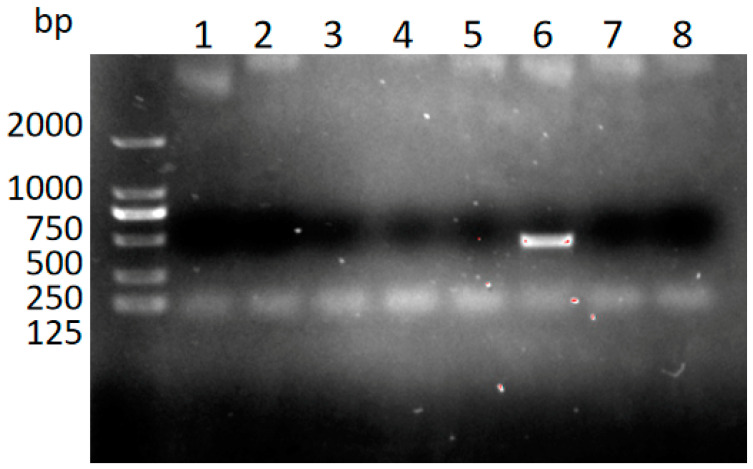
Detection of *T. gondii* in blood samples from different stray cats. Lanes 1–8 represent the respective numbers of the stray cats.

The results indicated that cat No. 6 harbored the *T. gondii* DNA (Figure 5). The sequence of the 5.8S rRNA gene in the positive blood sample from the cat was identical to that found in the infected kangaroos.

### 3.6. Treatment Effect of SDZ and PYR

Before the identification of the pathogen, SDZ and acetylspiramycin were, respectively, administered to the sick kangaroos at the initial stage of the disease, but this resulted in little relief of their clinical signs. However, after confirming the presence of *T. gondii* in the kangaroos and orally administering a combination of SDZ at 50 mg/kg and PYR at 1 mg/kg to each animal for 5 days, the clinical signs of the infected red kangaroos were significantly alleviated. This supported the correct diagnosis of toxoplasmosis.

## 4. Discussion

Kangaroos live mainly in the Australian continent and in parts of Papua New Guinea. They are a symbol of Australia and were extensively exported to other countries as one of the captive ornamental marsupials in zoos and nature reserves. However, due to their food preference for small grasses near the ground and fungi, they are more susceptible to being infected by pathogens scattered in the environment, including *T. gondii*. In addition, wild animal parks are located far from residential areas and outside the scope of human sanitary control, thus providing shelter for feral cats that can excrete oocysts to the environment, increasing the risk of infection.

To date, toxoplasmosis has been widely detected in Australasian imported kangaroos in Argentina, Japan, Chile, Germany, the USA, Brazil, etc. [19]. Recently, several cases of infected kangaroos have also been reported in Henan province of China [11,16]. In this study, we determined the infection of kangaroos in east China for the first time using nested PCR, IFAT, and HE and IHC staining. The IFAT results demonstrated the highest concentration of the tachyzoites in the cecum and lymph nodes of fatal cases, with a lower concentration of parasites in the brain. In contrast, previous studies have shown a high tropism of *T. gondii* for the myocardium in heart-transplanted human patients [20] and for brains in traumatic patients [21]. Another study also indicated that the highest potential for isolating *T. gondii* was achieved by inoculating mice with infected chicken brain tissue [22]. The IHC staining clearly indicated the presence of *T. gondii* in the heart, as well as in the liver and cecum, which exhibited similar staining patterns to those observed in the choroid plexus and frontal lobe of the brain in *T. gondii*-infected patients [23]. In Dubey et al.’s review, *T. gondii* DNA was detected in the heart of more than half of reported outbreaks of clinical toxoplasmosis [19]. Another article also described the most prevalent cysts in muscular and neural tissues [18]. In mice, intraperitoneal injection of *T. gondii* resulted in a higher number of parasites in the kidney, heart, and liver compared to those in other tissues (eye, muscle, and spleen) [24]. Feeding of oocysts in rats led to the detection of tissue cysts most often in the brain, skeletal muscles, heart, and kidneys, and the dose of oocysts had no effect on the distribution of tissue cysts [25]. However, in infected Mexican hairless pigs, no significant difference was observed in the tropism of *T. gondii* among the heart, tongue, and semimembranosus/gracilis muscles [26]. The tissue tropism of *T. gondii* needs further investigation.

Since the 5.8S rRNA gene sequence of *T. gondii* in the dead kangaroo showed 100% identity to a strain reported in stray cats in Shanghai in 2012 [17], we collected blood samples from eight stray cats in the vicinity of the kangaroos to explore the source for this infection. As expected, the 5.8S rRNA gene was positively detected in cat No. 6 and demonstrated the same sequence found in the dead kangaroos. This result suggests that the infection of captive kangaroos was closely related to the stray cats, indicating that the same strain might be spreading from these animals to captive ones. It is known that in Shanghai, the *T. gondii* infection rate is 9.1% (37/408) in dogs [27], 17.2% (25/145) in stray cats [17], 13.8% (160/1158) in slaughter pigs [28], 7.69% (3/39) in pet rabbits [29], 33.3% (4/12) in primates, 87.1% (27/31) in carnivores, and 10.5% (2/19) in herbivores from the zoological garden [30]. As for humans, previous data indicated an infection rate of 13.3% (119/893), and pet ownership, including cats, was considered as one of the main risk factors for *T. gondii* infection [31]. These data suggest an imperative need for the surveillance and control of *T. gondii* in east China.

It is intriguing that the western gray kangaroos (*M. fuliginosus ocydromus*) were kept in the same environment and lived together with the red ones, but only the red kangaroos developed clinical signs and even succumbed to death. This suggests that the red kangaroos were more sensitive to the epidemic strain of *T. gondii* than the gray ones. When 26 *T. gondii*-infected macropods in a zoo in Henan province were investigated, the proportion of red kangaroos (30.8%) was much higher than that of the gray ones (3.8%), as reported by Yang et al. [15]. Another previous study showed that even though 20% of plasma samples collected from 102 female western gray kangaroos tested positive for *T. gondii*, it did not affect the reproductive performance of the females [9], and it certainly did not result in the death of the infected animals. It is possible that the western gray kangaroos living in the same zoo as the red ones in this study were positive for anti-*T. gondii* antibodies. However, no sera were collected from them for testing. Further research is needed to clarify why and in what circumstances *T. gondii* is more fatal to red kangaroos than to gray ones.

As shown in Figure 3, scattered parasites shaped like crescents or petals were observed in the liver, spleen, lymph nodes, cecum, and brain of the dead kangaroos. In contrast, in the heart, the tachyzoites appeared to be condensed and contained a large number of progenies for an unknown reason. This trend was further confirmed by HE and IHC staining, as demonstrated in Figure 4. Since *T. gondii* is frequently cited as a cause of myocarditis in veterinary medicine [32], and given the ischemia observed in the heart, as shown in Figure 1c, we hypothesize that myocarditis caused by *T. gondii* may be the fatal factor leading to the kangaroos’ death. Some special proteins, such as Ldh1, expressed by the tachyzoites in these tissues, enhanced the acute parasite virulence [33] and, thus, may have been involved in the kangaroos’ death, as observed in the present study. However, further research needs to be carried out to support this hypothesis.

The popular drugs used to treat toxoplasmosis include PYR, SDZ, sulfamethazine (SMZ), acetylspiramycin, clindamycin, clarithromycin, azithromycin, and roxithromycin [34]. However, administration SDZ or acetylspiramycin solely to the infected kangaroos failed to treat the toxoplasmosis. This may suggest that the *T. gondii* clone CBa has developed drug resistance. This is the case for other *T. gondii* isolates, such as Ck3 and Pg1 [35], as well as RH-R^SDZ^ and ME-49-R^SDZ^ [36], which have demonstrated resistance to SDZ. Another possible reason could be the insufficiency of using only one inhibitor in the metabolic pathways of *T. gondii*. As is known, PYR and SDZ inhibit dihydrofolate reductase (DHFR) and dihydropteroate synthase (DHPS), respectively [37]. Therefore, PYR plus SDZ has been the most widely used treatment method and has proven effective in treating acute toxoplasmosis [34]. This combination also significantly alleviated the clinical signs of the infected animals in the present study.

## 5. Conclusions

In the present study, infection with *T. gondii* in three dead kangaroos was confirmed by nested PCR, IFAT, and IHC staining, which indicated the spread of the parasites within the organs of the infected animals. The source of infection was probably stray cats, as the same strain of *T. gondii* found in kangaroos was also detected in feline blood. This study underscores the high lethality of *T. gondii* in red kangaroos and the contamination of the environment by oocysts excreted by stray cats, thereby emphasizing the need for surveillance of *T. gondii* in the environment and implementing effective control measures for stray cats in zoos.

## Data Availability

The original contributions presented in the study are included in the article, further inquiries can be directed to the corresponding authors.
